# Role ambiguity and nursing interns’ achievement of clinical rotation goals: a correlational study

**DOI:** 10.1186/s12912-025-03842-y

**Published:** 2025-09-10

**Authors:** Manal Mohammed Ahmed Abdelaziz, Manal Saleh Moustafa Saleh, Zeinab Mohammed Aysha, Rehab Abd El-Moneim Abou Shaheen

**Affiliations:** 1 Nursing Department, North Private College of Nursing, Arar, Saudi Arabia; 2 Nursing Administration, Faculty of Nursing, Tanta University, Tanta, Egypt; 3 Department of Nursing Sciences, College of Applied Medical Science, Shaqra University, Shaqra, Saudi Arabia; 4 Nursing Administration, Faculty of Nursing, Zagazig University, Zagazig, Egypt; 5 Critical Care Nursing and Emergency, COHS Al Rayan National Colleges, Al Madina Al Monawara, Saudi Arabia; 6 Critical Care Nursing and Emergency, Faculty of Nursing, Tanta University, Tanta, Egypt; 7 Nursing Administration Department, Faculty of Nursing, Tanta University, Tanta, Egypt

**Keywords:** Internship year, Clinical rotation, Nursing interns, Role ambiguity

## Abstract

**Background:**

Nursing interns frequently encounter role ambiguity due to a mismatch between their expectations of the professional nursing role and the actual responsibilities they face in clinical settings. While clinical rotations during the internship year are intended to enhance clinical confidence and competence, such ambiguity can undermine these goals.

**Objective:**

To examine the relationship between internship clinical rotation and role ambiguity among nursing interns.

**Methods:**

A descriptive correlational study was conducted at Tanta University Hospitals, including six intensive care units, operating rooms, and general surgery departments. The study included all nursing interns (*n* = 900) enrolled in the internship year. Two tools were used to collect data: **Tool I**: Internship Clinical Rotation Fulfillment Questionnaire. **Tool II**: Role Ambiguity Questionnaire.

**Results:**

Less than half of the nursing interns reported that clinical rotations fulfilled their intended goals, while approximately one-quarter indicated only moderate fulfillment. Interns expressed significant ambiguity regarding their role definition, role performance, training, and psychosocial support. A significant positive correlation (*r* = 0.380, *p* < 0.001) was found, reflecting that lower role ambiguity (reverse-scored) correlated with higher goal achievement.

**Conclusion:**

The findings suggest that internship clinical rotations did not fully achieve their goals, and nursing interns experienced high levels of role ambiguity across multiple domains.

**Recommendations:**

It is recommended that structured orientation programs be implemented for nursing interns, outlining job roles, hospital policies, and regulations. Additionally, clinical nurse educators should be trained in effective supervision practices, and consistent performance feedback should be provided to interns throughout their rotations.

**Clinical trial:**

Not applicable.

## Introduction

The internship year represents a critical transition period during which nursing students evolve into professional practitioners through clinical rotations in specialized units, such as ICUs and operating rooms [1, 2]. However, this transition is frequently complicated by role ambiguity, a mismatch between interns’ expectations of their professional roles and the realities of clinical practice [3]. The goal of clinical rotation is to provide nursing interns with opportunities to learn in multiple patient care settings, receive appropriate guidance and instruction, and foster the development of clinical competencies and professionalism, which are essential to successfully fulfilling the role and responsibilities of a registered professional nurse [4, 5].

The competencies that make up the National Competency Standards for registered nurses are organized into four domains of nursing practice, professional and ethical practice, critical thinking and analysis, management of care, and enabling. Mostly clinical rotations during the internship year are designed to increase the clinical confidence and competence of nursing interns. Firstly, it should enhance professional and ethical practice, which requires demonstration of a satisfactory knowledge base, accountability for practice, functioning by legislation affecting nursing and health care, and the protection of individual and group rights. Secondly, enhance critical thinking and analysis related to self-appraisal, professional development, and the value of evidence and research for practice [6].

Thirdly, enhancing management of the care domain relates to the coordination, organization, and provision of nursing care that includes the assessment of individuals/ groups, planning, implementation, and evaluation of care. Fourthly, enhance enabling domain relates to establishing, sustaining, and concluding professional relationships with individuals/groups. Also, enhance competencies that relate to nurses’ understanding of their contribution to the interdisciplinary health care team [6]. Role ambiguity is one of the major nursing interns’ stressors in an internship year. Usually nursing interns experience reality shock because of the conflict between their expectations of the nursing role and the reality of the actual role in the work setting [7]. Their vague and unclear role expectation and uncertainty about what is expected of them led to their role ambiguity. There are two sources of ambiguity: objective and subjective ambiguity. Objective ambiguity arises from the lack of training and the lack of information needed for role definition and role performance. Subjective ambiguity is related to the social and psychological aspects of role performance [8, 9].

Nursing interns’ ambiguity related to role definition may result from a lack of clear, consistent information required for adequate performance and a lack of clear responsibilities for them to be able to carry out professional and ethical practices. Ambiguity related to role performance may result from insufficient information about work objectives, a lack of adequate supervision, and the absence of an orientation program to perform tasks. Ambiguity related to role training may result from insufficient training and experience to carry out their duties properly. Ambiguity related to social and psychological aspects, this more traumatic adjustment often correlated with inadequate and insufficient functional and emotional support, lack of practice experience and confidence, insecurities in communicating, and lack of support for the enactment of their professional practice values [10].

Existing research on nursing interns’ role ambiguity has primarily followed two approaches: (1) qualitative investigations of subjective experiences (e.g., Ismail & Gashgari’s [2] phenomenological study of NICU interns), and (2) small-scale correlational studies in general ward settings (e.g., Zhang et al., [11] analysis of 175 participants). The current study significantly extends this literature through three key contributions: **First**, by examining 900 interns across six distinct ICU specialties, we overcome the sample size and setting limitations identified by Serafin et al. [12]. **Second**, we provide the first quantitative analysis of how role ambiguity specifically impacts clinical rotation goal achievement. **Third**, we introduce a novel theoretical integration of Role Theory [13] with Benner’s clinical competency framework [14] to explain why ambiguity persists differently across specialty units.

### Significance of the study

During the internship year, many nurse interns feel uncomfortable and inadequate as their skill levels do not match their expectations of the role and responsibilities of practicing as a registered nurse. In addition, hospital administrators expect nurse interns to be competent to function and take responsibilities in nursing service at the time of graduation with adequate clinical and patient management skills to cover the shortage and decrease the workload on experienced staff [11]. Nursing intern’s clear role expectations about what is expected of them may help to achieve the goals of their clinical rotation and accept them develop many skills and competencies. So, this study will explore the influence of role ambiguity on nursing interns’ achievement of clinical rotation goals.

### Aim of the study

Explore the influence of role ambiguity on nursing interns’ achievement of clinical rotation goals. The study seeks to answer the following research questions:

1. What is the level of role ambiguity perceived by nursing interns during their clinical rotations?
2. How does role ambiguity influence nursing interns’ achievement of clinical rotation goals?

### Subjects and method

#### Study design

A descriptive correlational design was utilized to explore the relationship between role ambiguity and the achievement of clinical rotation goals among nursing interns. This design was appropriate for the nature of the research problem, as it enables the identification of relationships between variables using survey methods, especially when sufficient prior information exists. It allowed for systematic data collection and analysis regarding interns’ perceptions without manipulating the study environment.

#### Setting

The study was conducted at the Intensive Care Units (ICUs) and operating rooms of Tanta University Emergency Hospital (239 beds) and Main University Hospitals (927 beds), including: Medical ICU (18 beds), Anesthetic ICU (15 beds), Neurological ICU (18 beds), Neonatal ICU (22 beds), Pediatric ICU (7 beds), Cardiac ICU (23 beds) and Obstetric and General Surgery Operating Rooms.

### Sampling technique

A census total population sampling method was employed, whereby all nursing interns enrolled in the internship year 2024/2025 at the Faculty of Nursing, Tanta University (*n* = 900) during the data collection period were invited to participate. This approach ensured complete representation of the study population, minimized sampling bias, and allowed population‑level conclusions within the study context. The total population was surveyed because the size was manageable, participation could be integrated into interns scheduled clinical shifts, and this maximized statistical power. A post hoc power analysis, using observed effect sizes and α = 0.05, indicated greater than 0.90 power for detecting small‑to‑moderate correlations, supporting the robustness of the findings.

#### Study tools

Two tools were used to collect the data.

#### Tool I: Internship clinical rotation fulfillment questionnaire

This scale was established by Abdelaziz et al. (2016) [15]. **Part 1**: Demographic data (sex, age, experience, unit, and educational level). **Part 2**: It comprises four domains: **Role Definition** (thirteen items), e.g., “Maintain standards of professional code of ethics, provide opportunities to learn in multiple patient care settings, and develop the competencies necessary for professional practice.”; **Role Performance** (twelve items) e.g., “improve ability to carry out the duties and responsibilities of the nursing role, demonstrate commitment to continuing professional development, and provide opportunities to use critical thinking skills as part of problem solving and decision making.” **Role Training** (eleven items); e.g., “Develop abilities to work with an interdisciplinary team, provide opportunities to identify training needs in different clinical area, and Provide opportunities to use reflective practice as a part of daily work.“, and **Social and Psychological Aspects** (nine items) e.g.,” Identify areas of personal strength, weakness, and goals for nursing interns, provide opportunities to communicate with professionals, clients, and the public, and develop collaboration skills with health care providers.” Responses were rated on a five-point Likert scale: Strongly Agree = 5, Agree = 4, Neutral = 3, Disagree = 2, Strongly Disagree = 1. Fulfillment scores were classified as: Good (High) > 75%; Fair (Moderate) 60–75%; Poor (Low) < 60%.

#### Tool II: Role ambiguity questionnaire

Also developed by Abdelaziz et al. (2016) [15], used to assess the extent of role ambiguity across four dimensions: **Role Definition Ambiguity** (8 items) e.g., “Lack of authority to carry out job assignment, lack of clarity of job description and responsibilities, and lack of clear information needed to carry out my job.“. **Role Performance Ambiguity** (10 items) e.g., “Lack of appropriate supervision, inability to answer questions for patients or families, and workloads interfere with the quality of care provided.” **Role Training Ambiguity** (10 items) e.g., “inability to assess patient nutrition and fluid balance, inability to administer medications, and inability to follow infection control measure.” **Social and Psychological Ambiguity** (9 items) e.g., “Lack of support from the multidisciplinary team, lack of constructive feedback from registered nurses on the ward/unit, and lack of support from family during work role change”. Responses were rated on a five-point Likert scale: Strongly Agree = 1, Agree = 2, Neutral = 3, Disagree = 4, Strongly Disagree = 5. Ambiguity levels were classified as: <60%: High ambiguity, 60–80%: Moderate ambiguity and 80%: Low ambiguity.

## Methodology

### Permissions and ethical considerations

Ethical approval was obtained from the Scientific Research Ethics Committee at Tanta University’s Faculty of Nursing with approval code (6334). The first section of the paper contained all the information required for the study. A statement on the purpose and scope of the study was included in the questionnaire. Before starting their response to the document, each participant who selected the term consents to provide their informed consent. The respondents were assured that their replies would remain private and confidential, that their participation was voluntary, and that their absence would not have any detrimental effects on their grades or results. By the Helsinki Declaration’s requirements, participants have provided their informed consent. Participants were found to have the freedom to leave the study at any moment.

### Pilot study

A pilot study was conducted with 10% of the study sample to test the clarity and applicability of the tools. Data from the pilot study were used to refine the tools before the main data collection.

### Data collection phase

Data collection was conducted using two validated instruments: **Tool (I)**, the Internship Clinical Rotation Questionnaire, and **Tool (II)** the Role Ambiguity Questionnaire. The process was cross-sectional, designed to capture nursing interns’ perceptions during their clinical rotation period across different intensive care units. Data was collected in a single phase during the internship year. The researcher distributed the questionnaires to the interns in small groups during their scheduled shifts, ensuring minimal disruption to their clinical responsibilities. The interns completed the questionnaires in the presence of the researcher to clarify any doubts and ensure all items were fully answered. **Tool (I)** assessed the extent to which interns perceived the fulfillment of clinical rotation goals across four domains: professional role definition, role performance, role training, and social and psychological aspects. **Tool (II)** measured perceived role ambiguity across parallel domains. The full data collection process was conducted over three months from April to June 2024. This tool was validated by a panel of experts, and its reliability was confirmed using Cronbach’s Alpha (0.984 for the Internship Clinical Rotation Questionnaire and 0.955 for the Role Ambiguity Questionnaire, indicating high internal consistency.

### Statistical analysis

Data was analyzed using IBM SPSS Statistics (Version 22). Statistical significance was set at *p* < 0.05. Descriptive statistics (mean, standard deviation, range) were used for quantitative data. independent samples t-tests with df = 898, one‑way ANOVA with their df, plus effect sizes, followed by Tukey’s HSD post hoc tests, *p* ≤ 0.05. r) assessed the strength of relationships between variables.

## Result

**Table 1:** Mean score of nursing interns clinical rotation fulfillment according to their characteristics (*n* = 900)

Characteristics	*N*	Mean (SD)	Test Statistic (df)	*p*-value	Effect size
Age group					
< 22	664	137.75 (34.84)	t(898) = 0.023	0.982	d = 0.00
≥ 22	236	137.62 (26.49)			
Marital status					
Single	681	137.62 (34.82)	t(898) =-0.044	0.965	d = 0.01
Married	219	137.85 (30.05)			
Sex					
Male	101	144.28 (22.60)	t(898) = 0.901	0.369	d = 0.23
Female	799	136.89 (33.82)			
Number of children					
0	681	136.84 (33.25)	t(898) =-0.594	0.553	d = 0.11
≥ 1	219	140.44 (31.53)			
Previous qualification					
Technical Health Institute	73	134.92 (33.97)	t(898) =-0.320	0.749	d = 0.09
General Secondary School	827	137.97 (32.78)			
Outside training					
Yes	175	134.16 (33.63)	t(898) = -0.672	0.503	d = 0.13
No	725	138.57 (32.64)			
Medical ICU	163	133.21 (35.68)	F(8, 891) = 2.202	0.030*	ηp² = 0.02
Neurological ICU	95	142.59 (27.08)			
Pediatric ICU	130	142.22 (34.86)			
Gynecology	73	133.69 (33.70)			
Anesthetic ICU	135	151.79 (32.29)			
Neonatal ICU	101	142.56 (29.04)			
Cardiac ICU	85	122.27 (20.45)			
General Surgery	68	114.25 (39.86)			
Other	50	147.22 (21.14)			
Graduation level					
Excellent	203	140.31 (31.34)	F(3, 896) = 0.112	0.953	ηp² < 0.001
Very good	613	137.17 (33.01)			
Good	79	135.21 (37.59)			
Satisfactory	5	140.00 (-)			

N.B. *significant, p value ≤ 0.05

Table 1 indicates that there were no statistically significant differences in clinical rotation fulfillment scores across most demographic and training characteristics levels (*p* > 0.05). However, A statistically significant difference was observed for the Department variable (F (8, 891) = 2.20, *p* = 0.030, ηp² = 0.02, small effect size). Post hoc Tukey’s HSD tests indicated that interns in the Anesthetic ICU (M = 151.79, SD = 32.29) reported significantly higher fulfillment scores than those in the Cardiac ICU (M = 122.27, SD = 20.45, *p* = 0.008, d = 0.45) and General Surgery (M = 114.25, SD = 39.86, *p* = 0.002, d = 0.45), reflecting medium‑sized practical differences. No other departmental comparisons were statistically significant.

**Fig. 1:**
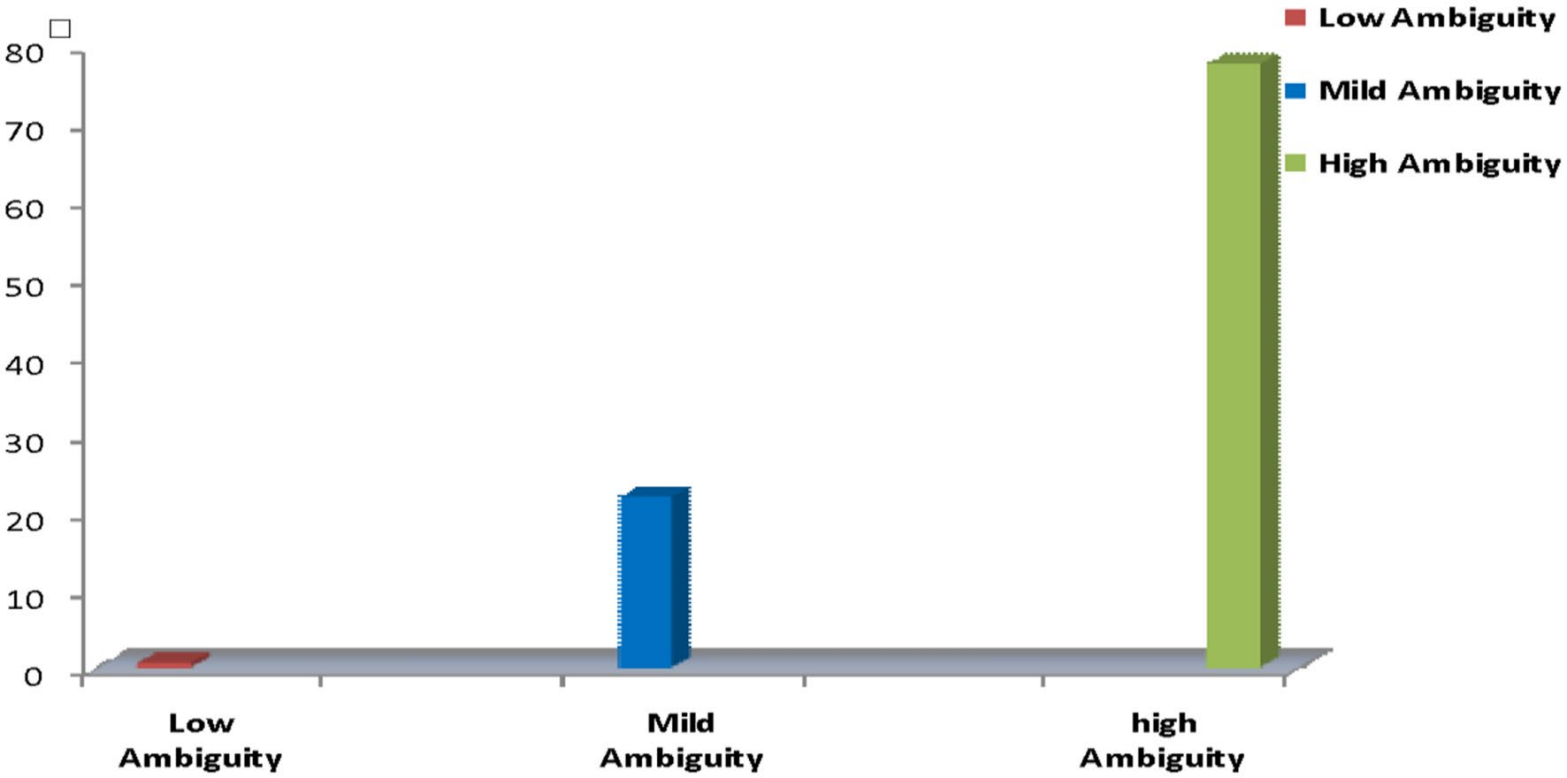
Nursing interns’ levels of total role ambiguity

Figure 1 shows the nursing intern’s levels of total role ambiguity. The majority (77.50%) of nursing interns showed high ambiguity, 22.88% showed mild ambiguity, and 0.63% showed no ambiguity of the total role.

**Fig. 2:**
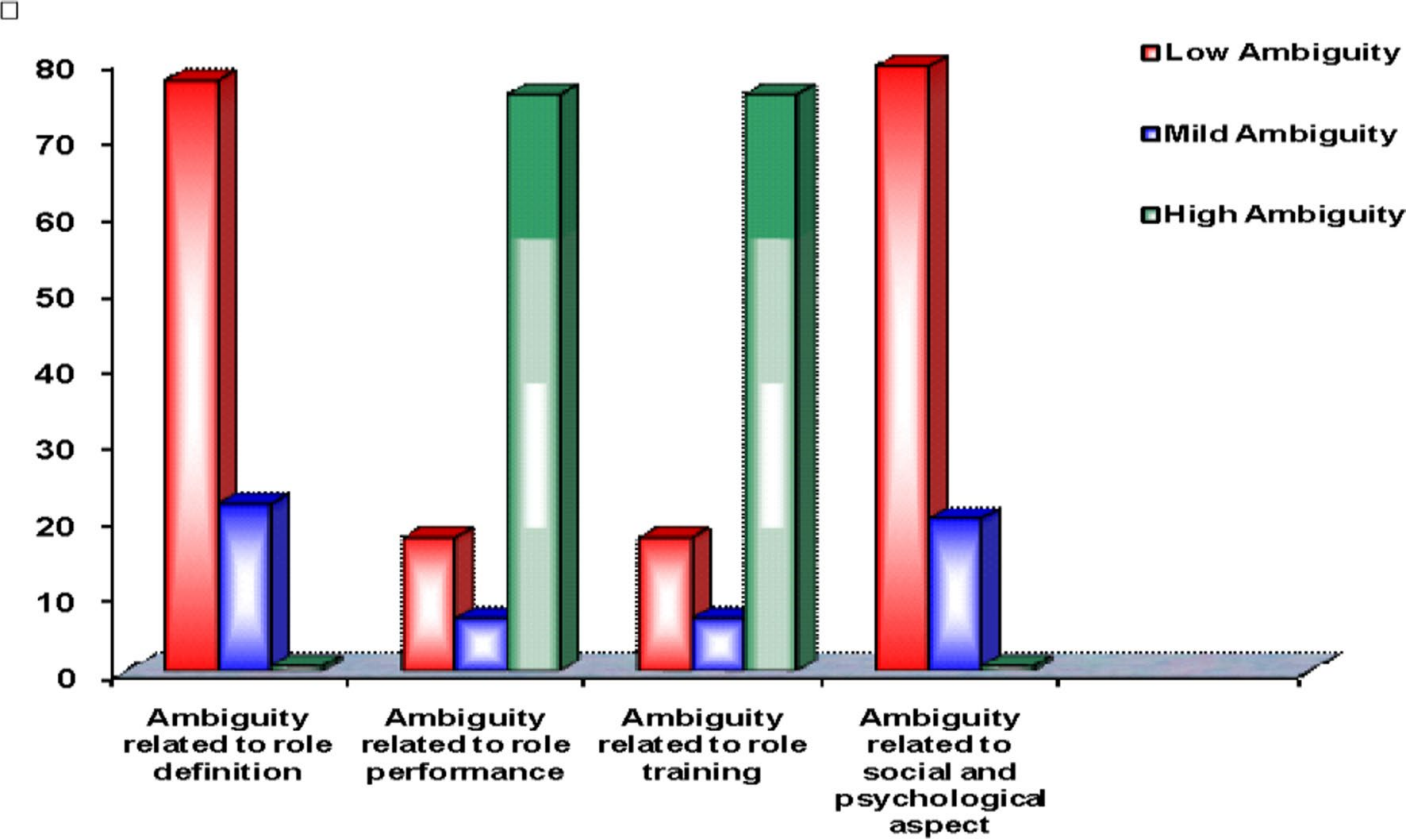
Nursing interns’ levels of different attributes of role ambiguity

Figure 2 shows nursing interns’ levels of different attributes of role ambiguity. An equal percentage of nursing interns (75.63%) showed ambiguity related to their role performance and role training. While about (21.88%) showed mild ambiguity of their role definition, the social and psychological aspects of their role. Nursing interns (79.38%) showed no ambiguity related to their social and psychological aspects and role definition.

**Table 2:** Mean score of nursing interns’ role ambiguity according to their characteristics. (*n* = 900)

Characteristics	*N*	Mean (SD)	Test Statistic (df)	*p*-value	Effect size
Age group					
< 22	664	89.01 (27.74)	t(898) = 0.878	0.381	d = 0.17
≥ 22	236	84.86 (21.75)			
Marital status					
Single Married	681 219	85.16 (24.31) 91.65 (28.53)	t(898) =-–1.549	0.123	d = 0.25
Sex					
Male	101	93.94 (22.53)	t(898) = 1.032	0.304	d = 0.27
Female	799	87.16 (26.71)			
Number of children					
0	681	89.58 (26.07)	t(898) =- 1.411	0.160	d = 0.26
≥ 1	219	82.77 (26.68)			
Previous qualification					
Technical Health Institute	73	92.31 (29.06)	t(898) =- 0.627	0.532	d = 0.17
General Secondary School	827	87.53 (26.11)			
Outside training					
Yes	175	92.48 (26.89)	t(898) = 1.077	0.283	d = 0.21
No	725	86.82 (26.14)			
Medical ICU	163	82.72 (28.64)	F(8, 891) = 2.870	0.005*	ηp² = 0.03
Neurological ICU	95	74.24 (28.11)			
Pediatric ICU	130	96.30 (27.76)			
Gynecology	73	86.23 (21.44)			
Anesthetic ICU	135	99.58 (20.75)			
Neonatal ICU	101	94.94 (20.20)			
Cardiac ICU	85	83.20 (30.64)			
General Surgery	68	70.58 (21.77)			
Other	50	97.33 (16.38)			
Graduation level			F(3, 896) = 0.562	0.641	ηp² < 0.001
Excellent	203	92.28 (25.35)			
Very good	613	86.39 (26.48)			
Good	79	89.64 (28.51)			
Satisfactory	5	74.00 (—)			

N.B *significant, p value ≤ 0.05

Table 2 indicates that role ambiguity scores were not statistically significant (*p* > 0.05) across most characteristics, including age group, marital status, sex, number of children, previous qualification, outside training, and graduation level, and effect sizes for these comparisons were generally small (d < 0.30), indicating negligible practical importance.

A notable exception was the department variable, for which the one-way ANOVA indicated a statistically significant difference in role ambiguity scores (*F* (8, 891) = 2.87, *p* = 0.005, ηp² = 0.03, small effect size). Post hoc Tukey’s HSD tests (not shown in the table) revealed that interns in the Anesthetic ICU (M = 99.58, SD = 20.75) and Pediatric ICU (M = 96.30, SD = 27.76) reported significantly higher ambiguity scores than those in the General Surgery unit (M = 70.58, SD = 21.77) and Neurological ICU (M = 74.24, SD = 28.11).

**Table 3:** Correlations between internship clinical rotation subscales and nursing interns’ role ambiguity subscales

Internship clinical rotation subscales							
Role ambiguity subscales	Role definition			Role performance	Role training	Social and psychological aspects.	Total clinical rotation
Ambiguity related to role definition	**r**		0.325	0.334	0.291	0.360	
	**P-value**		< 0.001*	< 0.001*	< 0.001*	< 0.001*	
Ambiguity related to role performance	**r**		0.264	0.347	0.287	0.305	
	**P-value**		0.001*	< 0.001*	< 0.001*	< 0.001*	
Ambiguity related to role training	**r**		0.261	0.162	0.205	0.201	
	**P-value**		0.001*	0.041*	0.009*	0.011*	
Ambiguity related to the social and psychological aspects	**r**		0.354	0.311	0.282	0.360	
	**P-value**		< 0.001*	< 0.001*	< 0.001*	< 0.001*	
Total Role	**r**						0.380
Ambiguity	**P-value**						< 0.001*

N.B *significant, p value < 0.001*

Table 3 A statistically significant positive correlation was found between the total Internship Clinical Rotation Fulfillment score and the total Role Ambiguity score (*r* = 0.380, *p* < 0.001). Additional significant correlations were observed between corresponding and cross‑domain subscales.

## Discussion

The internship year is a pivotal transition from nursing student to professional practice. In this study, 55.2% of nursing interns reported that their clinical rotations did not meet intended goals, with the lowest fulfillment scores observed in the role definition domain. This indicates that many interns lacked sufficient opportunities to develop essential professional and ethical behaviors, as well as skills in critical thinking, analytical reasoning, planning, care management, and leadership. Without clear objectives, adequate supervision, and structured orientation, interns were unable to effectively assess their progress or fully understand their responsibilities. These structural deficits are consistent with the elevated role ambiguity scores observed in this cohort, underscoring the need for comprehensive orientation programs and consistent guidance throughout the internship.

Evidence from [10] underscores the importance of structured support mechanisms, continuous evaluation, consistent mentorship, and comprehensive orientation in enhancing nursing interns’ preparedness and building professional confidence [16]. Similarly [17], identified persistent challenges in autonomy and readiness during the transition period, with inadequate supervisory support prolonging the “transition shock” phase marked by uncertainty, role ambiguity, and stress. From an organizational perspective [18], cautioned that job rotation, while intended to broaden experience, may lead to fatigue, task redundancy, and misalignment with institutional goals, reducing job satisfaction [19]. further noted that poorly timed or misaligned orientation can undermine role transition and jeopardize patient safety, while [20] highlighted the psychological strain of frequent ward rotations, reinforcing the need for emotional resilience. The negative impact of inadequate orientation is supported by [21, 22]who reported dissatisfaction rates of 16% and 8.6%, respectively, among new graduates, often linked to thoughts of career change. And [23] found that educational intervention proved to be a highly effective strategy for enhancing nursing students’ outcomes. By contrast, [24] found most interns felt prepared for clinical duties, suggesting that program effectiveness is shaped by contextual factors such as structure, institutional support, and regional standards.

In the present study, there was a statistically significant, moderate positive correlation between overall internship clinical rotation fulfillment scores and overall role ambiguity scores (*r* = 0.380, *p* < 0.001). This finding indicates that interns who perceived lower fulfillment of clinical rotation goals also tended to report higher levels of role ambiguity. The relationship was consistent across corresponding and cross-domain subscales, suggesting that deficiencies in one aspect of the rotation (e.g., role definition) are likely to be linked with ambiguity in other domains (e.g., role performance, role training, or psychosocial aspects).

Araújo et al. [25] reported a negative correlation between role ambiguity and internship satisfaction, noting that unclear responsibilities reduce engagement, satisfaction, and smooth transition. Similarly [26], identified a mismatch between the clinical experiences of new graduates and those they considered essential for preparedness, underscoring the need to align internships with competency requirements [27]. linked role ambiguity to insufficient information and weak integration into the social and psychological aspects of nursing practice, factors which, if unaddressed, can erode professional identity and belonging. In agreement [28, 29], found that clear role expectations enhance perceived competence, confidence, and team performance.

The current study revealed that most nursing interns reported high levels of ambiguity in role definition. They cited a lack of orientation regarding ethical standards, professional conduct, and institutional hierarchy. Additionally, they lacked exposure to professional codes of ethics and did not receive clear guidance to help develop the necessary competencies for professional nursing practice. While interns are responsible for their professional development, they require structured support [30, 31]. emphasized that nursing professional development is a lifelong process involving active participation in educational activities that maintain and enhance competence [32, 33]. similarly found that the orientation program failed to provide basic and general information about the internship year [34]. noted that preceptors, while expected to support new graduates, often manage full patient loads without compensation for their training responsibilities. This dual burden compromises both patient care and the professional development of interns.

Findings also revealed a significant correlation between role performance and role ambiguity. Many interns were expected to manage patient care responsibilities like their senior colleagues, but lacked access to experienced supervision. This resulted in increased emotional strain and inconsistency in care delivery. Interns also reported inadequate supervision and unclear organizational guidelines, which hindered their ability to perform clinical tasks effectively. Each ward, functioning as a specialized unit, imposes unique demands, requiring interns to adapt quickly to new team dynamics and workflows. Role ambiguity combined with role overload can impair decision-making and time management. Supporting studies by [32, 35] emphasized the significance of feedback-seeking behavior in qualifying the effects of role ambiguity. Likewise [36], associated feelings of inadequacy with insufficient procedural knowledge, contributing to a heightened fear of clinical errors.

Concerning role training, many interns reported insufficient opportunities to participate in educational and skill-development activities, particularly in patient and family communication. This lack of exposure limited their interpersonal skills and holistic care competencies. These findings are supported by [37]who found that interns were inadequately prepared for tasks like breaking bad news or managing patient anger [24, 38]. also stressed the importance of clinical support and job standardization in enhancing readiness for complex clinical scenarios. A significant positive correlation was also found between ambiguity in role training and interns’ reported challenges. Many struggled with basic care procedures and psychosocial support due to insufficient prior training.

As noted by [39], new graduates often experience role ambiguity due to ineffective communication, lack of knowledge, and fear of making mistakes [40, 41]. further highlighted the consequences of increased job demands and inadequate skills, emphasizing risks to both internal performance and patient safety. Finally, the study found a significant correlation between ambiguity in social and psychological aspects and the unfulfilled objectives of clinical rotation. Interns reported a lack of support and feedback from clinical supervisors and the wider healthcare team [42–44]. described how negative clinical environments and weak supervisory relationships lead to feelings of abandonment and reduced learning motivation. Feedback and appropriate supervision tailored to the intern’s stage of transition are essential for fostering professional growth and independence. In sum, while internship rotations serve as a crucial bridge between education and clinical practice, their effectiveness is undermined by inadequate preparation, lack of clarity, and insufficient support systems. Addressing these gaps through structured orientation, consistent supervision, and a well-defined role framework is essential to ensure a smoother transition and greater professional confidence among nursing interns [45]. 

## Conclusion

The findings of the present study revealed that nursing interns’ clinical rotations did not fulfill their intended goals, particularly in the areas of role definition, role performance, role training, and social and psychological aspects. This lack of fulfillment led to a high level of role ambiguity among nursing interns across all examined domains. Furthermore, a highly significant positive correlation was found between the total scores of internship clinical rotation subscales and the total scores of role ambiguity subscales. These results underscore the critical need for structured interventions aimed at clarifying role expectations and strengthening the support systems available to nursing interns during their clinical training.

## Limitations, future directions, and recommendations

### Limitations

Despite the valuable insights provided by this study, several limitations should be acknowledged. First, the research was conducted within a single institutional context, Tanta University Hospitals, which may limit the generalizability of the findings to other settings with different educational structures or clinical environments. Second, a cross-sectional design captures perceptions at a single point in time, thereby limiting the ability to assess longitudinal changes in role ambiguity or the impact of interventions over time. Third, reliance on self-reported data may introduce response bias, as participants might underreport or overreport their experiences due to social desirability or misunderstanding of questionnaire items. Finally, the absence of qualitative data restricts a deeper exploration of the subjective experiences and contextual nuances influencing role ambiguity among nursing interns.

### Future directions

To build upon the current findings, future research should consider longitudinal designs to examine the evolution of role ambiguity throughout the internship year and its longer-term effects on professional development and retention. Multi-site studies involving diverse academic and clinical institutions are recommended to enhance the generalizability of results and to identify context-specific barriers and facilitators to successful internship experiences. Incorporating qualitative methods such as interviews or focus groups would provide richer insights into the personal and organizational dynamics contributing to role ambiguity. Additionally, intervention studies are warranted to test the effectiveness of structured orientation programs, mentorship models, and competency-based role delineation, thereby establishing evidence-based strategies for improving clinical training outcomes.

### Recommendations

Based on the study’s findings and limitations, the following evidence-based recommendations are proposed to enhance internship effectiveness and reduce role ambiguity. The following recommendations are proposed:

1. Educational Institutions: Nursing faculties should develop comprehensive, standardized orientation programs tailored to each clinical rotation. These programs must clearly define interns’ roles, responsibilities, and expected competencies.
2. Clinical Supervision: Institutions should invest in training clinical educators and preceptors in evidence-based supervision strategies that promote autonomy, feedback, and psychosocial support for interns.
3. Policy Development: Clear job descriptions and structured role expectations should be formally established and communicated through institutional policies and educational materials such as booklets or digital platforms.
4. Support Structures: Hospitals should adopt mentorship models that pair interns with experienced nurses to provide ongoing support, evaluation, and skill development opportunities throughout the internship year.
5. Workload Management: Nurse managers must ensure that workloads assigned to nursing interns are appropriate to their competence level and promote skill development without contributing to burnout or role overload.
6. Feedback Systems: Regular, constructive feedback from multidisciplinary team members, including registered nurses and clinical nurse managers, should be institutionalized as a core component of the internship program.

### Implications for nursing management and leadership

The findings of this study have significant implications for nursing management and leadership, particularly in the areas of clinical education, workforce development, and organizational effectiveness. The high levels of role ambiguity reported among nursing interns underscore the need for nurse managers and leaders to critically evaluate the structure and delivery of internship programs within their institutions. First, nursing leadership must prioritize the development and implementation of clear role definitions and expectations for nursing interns. Ambiguity in clinical responsibilities can compromise not only the interns’ learning outcomes but also the quality and safety of patient care. Leaders should ensure that role clarity is established through well-documented job descriptions, orientation manuals, and communicated expectations at the outset of each clinical rotation.

Second, the study highlights the necessity of transformational leadership approaches in managing clinical training environments. Nurse leaders are encouraged to adopt supportive, mentoring roles that foster open communication, psychological safety, and professional growth among interns. Leadership styles that emphasize empowerment, recognition, and feedback are essential for building clinical confidence and fostering commitment among new graduates. Third, nursing managers must advocate for and allocate resources toward comprehensive orientation and preceptorship programs. These programs should be standardized across clinical units to ensure consistency in training and support. Managers should also collaborate with academic institutions to align educational objectives with clinical competencies and ensure that the transition from student to practitioner is seamless and supportive. Moreover, nurse leaders should implement mechanisms for continuous feedback and performance appraisal, which can help interns identify strengths, address weaknesses, and reduce the uncertainty associated with role ambiguity. Structured feedback systems promote a culture of accountability and continuous improvement, both of which are critical to professional development. Finally, the study suggests a need for leadership investment in interns’ well-being and resilience-building strategies. The emotional and psychological demands of the internship year necessitate supportive leadership practices that reduce stress, prevent burnout, and sustain intern motivation. Leadership training programs should include components on emotional intelligence, conflict resolution, and supportive supervision to equip nurse leaders with the skills required to nurture novice practitioners.

## Data Availability

The data supporting the findings of this study are available from the corresponding authors upon reasonable request.

## References

[1] Abd Elmenem Ibrahim A, Fathi Sleem W. Relationship between reality shock and career resilience among nursing interns’ students. Egypt J Health Care. 2021;12(3):214–25. 10.21608/ejhc.2021.189656.

[2] Ismail A, Gashgari R. Experiences of nursing interns in the neonatal intensive care unit in Saudi Arabia: a phenomenological study. Cureus. 2024;16(12):e74893. 10.7759/cureus.74893. 39742177 10.7759/cureus.74893 PMC11686417

[3] Akeel A, Kabee A. Nursing interns’ orientation program: a predictor of improving nursing interns’ knowledge about professional nursing practice. Egypt J Nurs Health Sci. 2023;4(4):243–73. 10.21608/ejnhs.2023.339486.

[4] Haruzivishe C, Macherera DM. Perceived readiness to practice among BSC honors in nursing graduates: implications for training. Open Access Libr J. 2021;8(4):1–12.

[5] Moustafa Saleh MS, Elsabahy HES. Integrating sustainability development education program in nursing to challenge practice among nursing interns in health care. J Nurs Manag. 2022;30(8):4419–29. 10.1111/jonm.13376. 36219534 10.1111/jonm.13869

[6] Lukewich J, Allard M, Ashley L, Aubrey-Bassler K, Bryant-Lukosius D, Klassen T, Wong ST. National competencies for registered nurses in primary care: a Delphi study. West J Nurs Res. 2020;42(12):1078–87. 10.1177/0193945920935590. 32615873 10.1177/0193945920935590 PMC7594255

[7] Popoola MN. Nurturing future nursing leaders: strategies for improved transition and retention of new graduate nurses [Master’s thesis]. San Francisco State University. Available online.

[8] Joo H, Lee YJ, Lee H. Factors affecting job satisfaction and turnover intention among newly graduated nurses in Korea. Global Health Nurs. 2022;12(1):69–77.

[9] Saleh MSM. The effect of an educational workshop on nurse interns towards setting priority of nursing care at Zagazig University Hospital in Egypt. IOSR J Nurs Health Sci. 2018;7(5 – 4):67.

[10] Sparks K, Cooper CL. Occupational differences in the work-strain relationship: towards the use of situation-specific models. Managerial, occupational and organizational stress research. Routledge; 2024. pp. 537–48.

[11] Zhang HL, Wu C, Yan JR, Liu JH, Wang P, Hu MY, et al. The relationship between role ambiguity, emotional exhaustion and work alienation among Chinese nurses two years after COVID-19 pandemic: a cross-sectional study. BMC Psychiatry. 2023;23(1):516. 10.1186/s12888-023-04923-5. 37464335 10.1186/s12888-023-04923-5 PMC10355025

[12] Serafin L, Pawlak N, Strząska-Kliś Z, Bobrowska A, Czarkowska-Pączek B. Novice nurses’ readiness to practice in an ICU: a qualitative study. Nurs Crit Care. 2022;27(1):10–8. 10.1111/nicc.12730. 33624431 10.1111/nicc.12603

[13] Kahn RL, Wolfe DM, Quinn RP, Snoek JD, Rosenthal RA. Organizational stress: studies in role conflict and ambiguity. Wiley; 1964.

[14] Benner P. From novice to expert: excellence and power in clinical nursing practice. Addison-Wesley; 1984.

[15] Abd-ELaziz M. Study relationship of internship clinical rotation and role ambiguity among nursing interns [Unpublished Master’s thesis]. Faculty of Nursing, Tanta University.

[16] Saleh MSM, Ata AA, Abd-Elhamid ZN, Eltahan AA, Dailah HG, Elsabahy HE. Building nursing leaders: the influence of entrepreneurial leadership program on nurse interns’ innovation and clinical performance. BMC Nurs. 2025;24(1):1. 40340834 10.1186/s12912-025-03100-1 PMC12063326

[17] Reebals C, Wood T, Markaki A. Transition to practice for new nurse graduates: barriers and mitigating strategies. West J Nurs Res. 2022;44(4):416–29. 33724088 10.1177/0193945921997925

[18] Hailu K. Effectiveness of job rotation practices: the case of Dashen Bank SC [Doctoral dissertation]. St. Mary’s University; 2019.

[19] Labrague LJ, De los Santos JAA. Transition shock and newly graduated nurses’ job outcomes and select patient outcomes: a cross-sectional study. J Nurs Adm Manag. 2020;28(5):1070–9. 10.1111/jonm.13014. 10.1111/jonm.13033 32315478

[20] Reynolds E. New graduate nurse transition to practice and retention in rural settings: a mixed methods study [Doctoral dissertation]. University of Ottawa; 2024.

[21] Mohammed BMA, Ahmed WAM. Evaluation of nurse interns’ satisfaction and hospital as an educational environment in nursing internship training program, Saudi Arabia. Saudi J Health Sci. 2020;9(1):22–9.

[22] Hallaran AJ, Edge DS, Almost J, Tregunno D. New nurses’ perceptions on transition to practice: a thematic analysis. Can J Nurs Res. 2023;55(1):126–36. 35068206 10.1177/08445621221074872 PMC9936430

[23] Alhowaymel FM, Saleh MSM, Taref NN, Abd-Elhamid ZN, Abaoud AF, Alenezi A, Elsabahy HES. Empowering future nurses: enhancing self-efficacy, satisfaction, and academic achievement through talent management educational intervention. BMC Nurs. 2025;24(1):875. 40624493 10.1186/s12912-025-03512-z PMC12235881

[24] El-Kashif ML, Elsaiad HSA, Eid Baker MF, Thabet HA. Effect of evidence-based practice program on internship students’ performance at the maternity nursing departments. Egypt J Health Care. 2023;14(3):883–903.

[25] Araújo AAC, de Godoy S, de Oliveira RM, Vedana KGG, de Sousa ÁFL, Wong TKS, Mendes IAC. Positive and negative aspects of psychological stress in clinical education in nursing: a scoping review. Nurse Educ Today. 2023;126:105821. 10.1016/j.nedt.2023.105821. 37080012 10.1016/j.nedt.2023.105821

[26] den Hertog R, Boshuizen HP. Learning professional knowledge: bachelor nursing students’ experiences in learning and knowledge quality outcomes in a competence-based curriculum. Vocations Learn. 2022;15(1):21–47.

[27] Baharum H, Ismail A, McKenna L, Mohamed Z, Ibrahim R, Hassan NH. Success factors in adaptation of newly graduated nurses: a scoping review. BMC Nurs. 2023;22(1):125. 10.1186/s12912-023-01359-2. 37069647 10.1186/s12912-023-01300-1 PMC10111715

[28] López-Ibort N, Cañete-Lairla MA, Gil-Lacruz AI, Gil-Lacruz M, Antoñanzas-Lombarte T. The quality of the supervisor–nurse relationship and its influence on nurses’ job satisfaction. Healthcare. 2021;9(10):1388. 10.3390/healthcare9101388. 34683067 10.3390/healthcare9101388 PMC8544584

[29] Saleh MSM, Ata AA, Abd-Elhamid ZN, Eltahan AA, Dailah HG, Elsabahy HE. Visionary leadership: the mediating role of organizational support on nurse interns’ creativity and organizational effectiveness. BMC Nurs. 2025;24(1):400. 10.1186/s12912-025-02951-y. 40211308 10.1186/s12912-025-02951-y PMC11987171

[30] Baker C, Cary AH, da Conceicao Bento M. Global standards for professional nursing education: the time is now. J Prof Nurs. 2021;37(1):86–92. 10.1016/j.profnurs.2020.06.002. 33674114 10.1016/j.profnurs.2020.10.001 PMC7571445

[31] EL-Housary N, Shabaan F, ELDemerdash S, Shokier M. Effect of educational program on head nurses’ coaching skills to manage novice nurses’ role ambiguity [Master’s thesis]. Tanta University, Faculty of Nursing.

[32] Abdel Wahab GHES, Ibrahem El-Sayed R, Ibrahiem Khereba WM, El Diasty NAG. Effectiveness of internship program as perceived by intern nurses and its relation to their professional role at technical nursing institutes. Port Said Sci J Nurs. 2021;8(1):255–74.

[33] Abdelhameed Ismail Hassan H, Mohamed Rashad Ebrahim R, Samir Abd El-Aziz Elsaiad H. The relation between role clarity and self-confidence as perceived by intern nurses. J Nurs Sci Benha Univ. 2025;6(1).

[34] Sumpter D, Blodgett N, Beard K, Howard V. Transforming nursing education in response to the future of nursing 2020–2030 report. Nurs Outlook. 2022;70(6):S20–31. 10.1016/j.outlook.2022.08.004. 36446537 10.1016/j.outlook.2022.02.007

[35] Zeng L, Chen Q, Fan S, Yi Q, An W, Liu H, et al. Factors influencing the professional identity of nursing interns: a cross-sectional study. BMC Nurs. 2022;21(1):200. 10.1186/s12912-022-01054-7. 35879704 10.1186/s12912-022-00983-2 PMC9310353

[36] Manolache M, Epuran G. The mediating impact of goal–role clarity on the relationship between feedback–seeking behavior and goal orientations with job satisfaction intrinsic cognitions and person–organization fit. Sustainability. 2023;15(17):12776. 10.3390/su151712776.

[37] Mazalová L, Gurková E, Štureková L. Nursing students’ perceived stress and clinical learning experience. Nurse Educ Pract. 2022;64:103457. 10.1016/j.nepr.2022.103457. 36182730 10.1016/j.nepr.2022.103457

[38] Lee YK, Ng CJ, Sim JH, Firdaus A, Foong CC, Hong WH, et al. Barriers to effective research supervision in clinical specialist training: experience from a medical school in Malaysia. Malaysian Family Physician. 2021;16(3):77–82. https://www.ncbi.nlm.nih.gov/pmc/articles/PMC8432808/. 34938395 10.51866/oa1222 PMC8680945

[39] Towner EC, East LS, Lea J. The experiences of new graduate nurses caring for the deteriorating patient in rural areas: an integrative review. Collegian. 2022;29(2):245–51. 10.1016/j.colegn.2021.09.005.

[40] Chang E, Hatcher D. Transitions in nursing - E-book: preparing for professional practice. Elsevier Health Sciences; 2024.

[41] Seery K, Barreda AA, Hein SG, Hiller JL. Retention strategies for online students: a systematic literature review. J Global Educ Res. 2021;5(1):72–84. 10.5038/2577-509X.5.1.1166.

[42] Kerr H, Rainey D. Addressing the current challenges of adopting evidence-based practice in nursing. Br J Nurs. 2021;30(16):970–4. 10.12968/bjon.2021.30.16.970. 34514831 10.12968/bjon.2021.30.16.970

[43] El-Sayed AAI, Abdelaliem SMF. Application of Kano model for optimizing the training system among nursing internship students: a mixed-method Egyptian study. BMC Nurs. 2023;22(1):316. 10.1186/s12912-023-01481-3. 37710268 10.1186/s12912-023-01485-5 PMC10500916

[44] Ambunya LO. Role conflict, role ambiguity and burnout among head teachers of public primary schools in Kakamega County, Kenya [Doctoral dissertation]. Masinde Muliro University of Science and Technology; 2020.

[45] Tuomikoski AM, Ruotsalainen H, Mikkonen K, Kääriäinen M. Nurses’ experiences of their competence at mentoring nursing students during clinical practice: a systematic review of qualitative studies. Nurse Educ Today. 2020;85:104258. 10.1016/j.nedt.2019.104258. 31830638 10.1016/j.nedt.2019.104258

